# Volvulus of the Transverse Colon Herniated Through Drain Site

**DOI:** 10.7759/cureus.34151

**Published:** 2023-01-24

**Authors:** Veda Prakash, Oseen Shaikh, Sagar Prakash, Chellappa Vijayakumar, Uday Kumbhar

**Affiliations:** 1 Surgery, Jawaharlal Institute of Postgraduate Medical Education and Research, Puducherry, IND

**Keywords:** intestinal obstruction, drain site, hernia, transverse colon, volvulus

## Abstract

Herniation of the transverse colon and volvulus of it occurring through the previous surgical drain site, presenting as intestinal obstruction, has never been reported. We present an 80-year-old female who complained of abdominal swelling for 10 years. She started developing pain abdomen for 10 days and obstipation for three days. Abdominal examination showed a tender mass in the right lumbar region, with all borders being distinct, and there was no cough impulse. There is a lower midline scar from the previous laparotomy and a small scar over the swelling (drain site). Imaging studies were diagnostic of large bowel obstruction due to the herniation and volvulus of the transverse colon through the previous surgical drain site. She underwent laparotomy, derotation of transverse colon with hernia reduction, and onlay meshplasty. She had an uneventful postoperative course and was discharged.

## Introduction

Transverse colon herniation can occur through a previous abdominal surgical scar. The transverse colon can undergo volvulus and may present with features of intestinal obstruction. Volvulus of the herniated transverse colon through the previous surgical drain site is never known to occur. No such case has been reported in the literature so far. The typical clinical presentations of the patient with transverse colon volvulus usually present with features of intestinal obstruction. Patients who develop obstructed incisional hernia also present with abdominal pain, abdominal mass, nausea, vomiting, and obstipation [1,2]. Imaging studies such as abdominal X-rays and computed tomography (CT) help diagnose such conditions. Patients with obstructed incisional hernia or transverse colon volvulus should undergo emergency laparotomy to relieve obstruction, resection of unviable bowel if any, and repair of fascial defect. Here we present an 80-year-old female with intestinal obstruction features due to herniation and volvulus of the transverse colon through the previous surgical drain site.

## Case presentation

An 80-year-old female presented to the emergency department with complaints of swelling in the right flank region for the last ten years. The swelling was gradually progressive in size. The swelling increased in size on straining and reduced partially on lying down. Now she has complained of intermittent colicky abdominal pain for ten days, abdominal distension, and obstipation for three days. There was no history of vomiting, fever, jaundice, or reduced urine output. She gave a history of abdominal hysterectomy 30 years back, with re-exploration in the immediate postoperative period, probably due to gossypiboma with drain placement in the right flank region.

At the presentation, she was dehydrated; her pulse rate was 110 beats per minute, blood pressure was 110/80 mm Hg. Abdominal examination revealed swelling of 20 cm x 20 cm over the right lumbar region, tender, and firm in consistency. All borders of the swelling were distinct and round. There was no cough impulse in the swelling. There is a lower midline scar from the previous laparotomy and a small scar over the swelling (drain site). Bowel sounds were exaggerated. Per rectal examination revealed collapsed rectum.

**Figure 1:**
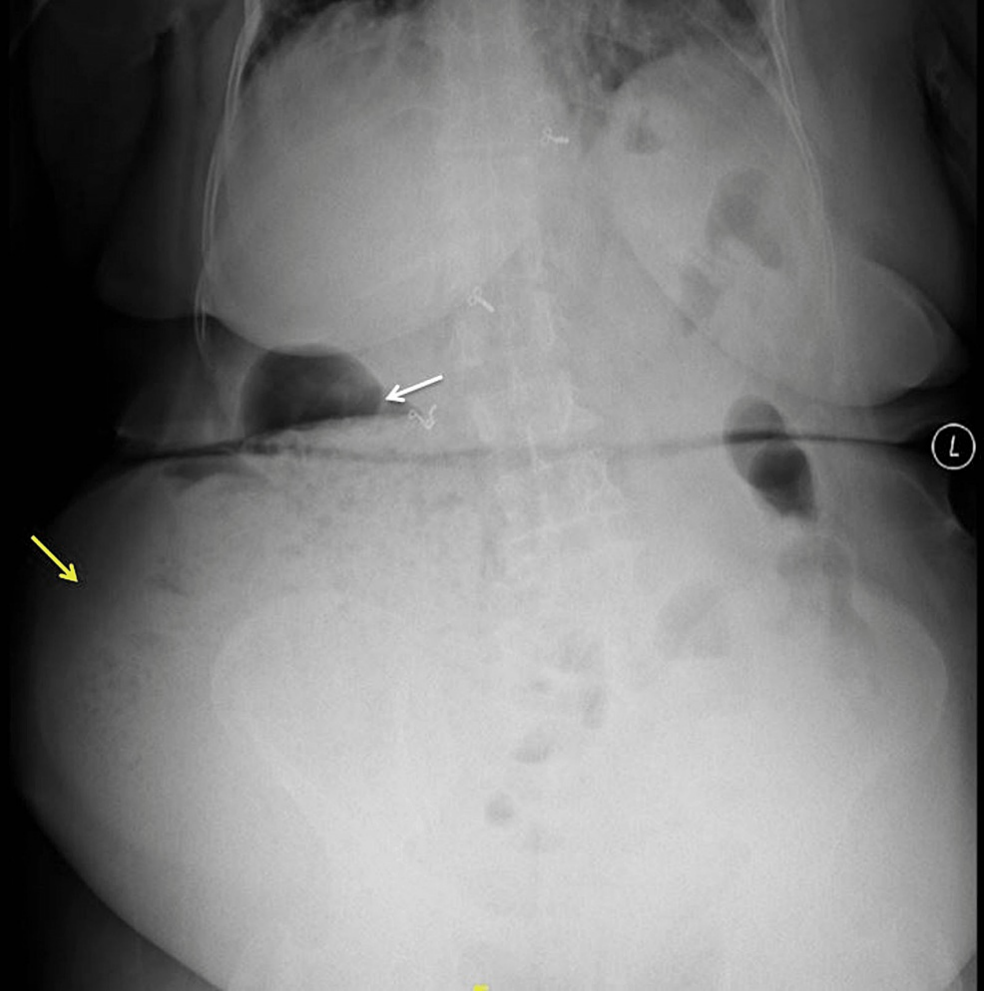
X-ray abdomen (erect view) showing air-fluid level (white arrow) and herniated and obstructed bowel loop (yellow arrow).

Blood investigations showed hemoglobin of 13.4 g/dL, leukocytosis with 15,000 cells/mm^3^, and normal platelet count. The liver function test (LFT) and renal function test (RFT) were within normal limits. Arterial blood gases were within normal limits. X-ray abdomen showed a few dilated bowel loops on the right side of the abdomen with air-fluid levels. There was no air under the diaphragm (Figure 1).

**Figure 2:**
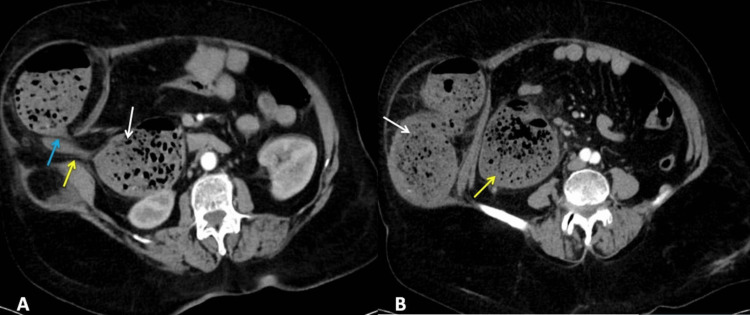
Computed tomography (axial view) showing (A) previous drain site (yellow arrow), grossly dilated right colon (white arrow), volvulus of the herniated transverse colon (blue arrow), (B) a grossly dilated segment of the transverse colon (white arrow) and grossly dilated right colon (yellow arrow).

Ultrasonography of the abdomen showed dilated content-filled bowel loops showing sluggish peristalsis in the right iliac fossa region. Rests of the solid organs were grossly normal. Contrast-enhanced CT abdomen showed herniation of the transverse colon through a defect in the right lateral abdominal wall with features of obstruction (Figures 2A, 2B).

**Figure 3:**
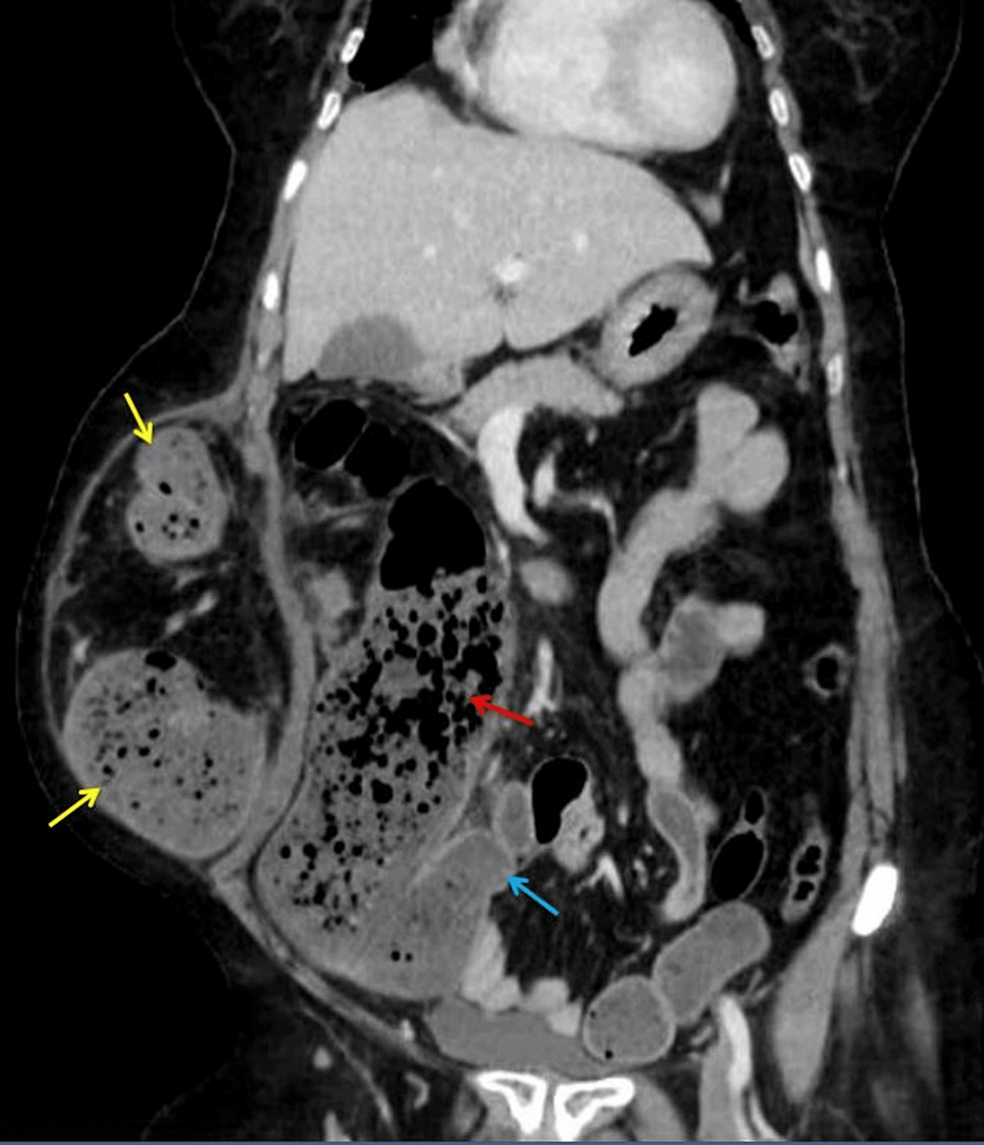
Computed tomography (coronal view) showing grossly dilated right colon (red arrow), the ileocecal junction (blue arrow), and herniated transverse colon volvulus at the previous drain site (yellow arrows).

There was evidence of volvulus of the transverse colon. There was no evidence of bowel gangrene in herniated bowel (Figure 3).

**Figure 4:**
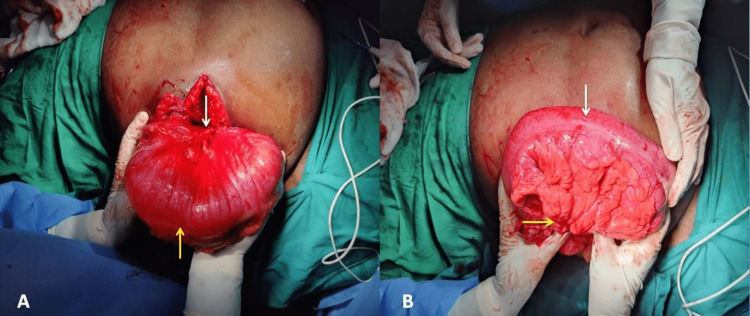
Intraoperative image showing (A) volvulus (white arrow-360-degree rotated) of the transverse colon (yellow arrow) and (B) derotated (yellow arrow) transverse colon (white arrow).

The patient underwent emergency surgical exploration. A transverse incision was taken over the swelling. Transverse colon and omental herniation were noted through a 4-cm defect in the lateral abdominal wall with 360-degree rotation of the transverse colon. No gangrenous changes were present. The transverse colon was derotated and pushed back into the abdomen (Figures 4A, 4B).

Partial omentectomy was done because of adhesions. Mesh was placed to reinforce the defect. She recovered well and was discharged 10 days post-surgery. The patient was followed for the next three months. She was completely asymptomatic and did not have a recurrence.

## Discussion

Hernias at previous surgical incisions are referred to as incisional hernias. Almost 3% to 11% of the patients who underwent abdominal surgery will develop an incisional hernia [3,4]. Hernias occurring through the previous surgical drain site are also known to occur and are usually considered a type of incisional hernia [2]. Volvulus of the large bowel is a rare entity and accounts for 3% to 5% of cases of intestinal obstruction. This can lead to closed-loop obstruction, gangrene, and perforation in neglected cases. The volvulus of the transverse colon is an infrequent entity and represents only 3% of all cases of colonic volvulus [5]. Transverse colon herniation occurring through a drain site is rare. Volvulus of the herniated transverse colon through the drain site has never been reported in the literature.

Multiple risk factors for incisional hernias are well recognized, such as obesity, poor nutrition, infection at the drain site, old age, and increased intra-abdominal pressure due to various causes like a chronic cough [3]. Risk factors for the development of transverse colon volvulus include redundancy and non-fixation of the colon, previous volvulus, distal colonic obstruction, adhesions, malposition of the colon during previous surgery, mobility of the right colon, inflammatory strictures and carcinoma, chronic constipation, Clostridium difficile associated pseudomembranous colitis and impaired intestinal motility in pregnancy [6,7]. Our patient was obese, with a history of laparotomy probably for Gossypiboma with drain placement.

Patients with transverse colon volvulus typically present with features of intestinal obstruction. Vomiting is an early feature in a patient with transverse colon volvulus due to compression of the duodenum by the transverse colon. Patients with obstructed incisional hernias will also have intestinal obstruction features and irreducible swelling in the abdominal wall. In the initial period, hyperactive bowel sounds may be present. These bowel sounds may be absent in the later period of the disease [8]. In our patient, vomiting was not an early feature, probably due to the long-standing herniation of the transverse colon through the drain site, which underwent volvulus, leading to acute intestinal obstruction.

Transverse colon volvulus diagnosis is challenging to make preoperatively. Transverse colon volvulus does not have characteristic radiological features as that of sigmoid colon volvulus. The diagnosis of transverse colon volvulus is usually made intraoperatively [9]. A plain x-ray abdomen may show a distended colonic loop with an air-fluid level. CT abdomen is usually indicated in a patient with suspected volvulus or obstructed incisional hernia. CT also will demonstrate the presence of bowel enhancement or any features of the development of bowel gangrene [10].

Management of obstructed incisional hernia is primarily surgical with immediate exploration, delaying which might result in gangrene or even perforation [11]. A synthetic mesh is routinely placed in case there is no gross contamination. Treatment of the transverse colon volvulus includes resection of the transverse colon, with primary anastomosis or creating a stoma. Compared with resection, simple detorsion of the bowel or untwisting with colopexy is associated with a higher recurrence rate or even death [12]. However, in our case, volvulus was present inside the hernia sac, and the neck of the hernia sac was very narrow. We performed a laparotomy, derotated herniated bowel loop, and reduced back in the abdomen.

## Conclusions

The literature has never reported transverse colon volvulus through the previous surgical drain site. The surgeon should be aware of such a rare condition for a patient presenting with irreducible abdominal swelling with features of intestinal obstruction. Preoperative imaging studies play an essential role in the diagnosis of such conditions and in planning surgical management. Transverse colon volvulus usually requires resection of the transverse colon. However, in patients with transverse colon volvulus through the drain site, the colon can be reduced back into the abdomen after derotation (if there is no evidence of bowel gangrene).
